# The impact of social insurance on health among middle-aged and older adults in rural China: a longitudinal study using a three-wave nationwide survey

**DOI:** 10.1186/s12889-020-09945-2

**Published:** 2020-12-01

**Authors:** Xinxin Ma, Takashi Oshio

**Affiliations:** 1 Faculty of Social Sciences, University of Toyama, 3190 Gofuku, Toyama, 930-8555 Japan; 2 Institute of Economic Research, Hitotsubashi University, 2-1 Naka, Kunitachi-shi, Tokyo, 186-8603 Japan

**Keywords:** Dynamic fixed-effects regression model, New rural social pension insurance, New rural cooperative medical scheme, Middle-aged and older adults, Rural China

## Abstract

**Background:**

Many studies have examined the impact of social insurance on health, but the results have generally been mixed, presumably because they have not fully addressed potential biases related to the study’s cross-sectional design. In this study, we conducted a longitudinal analysis to investigate how participation in two social insurance programs in China—the New Rural Social Pension Insurance (NRSPI) and the New Rural Cooperative Medical Scheme (NRCMS)—was associated with health outcomes among middle-aged and older adults in rural China.

**Methods:**

Using three-wave longitudinal data from the China Health and Retirement Longitudinal Study conducted in 2011, 2013, and 2015, we estimated the dynamic fixed-effects regression models to examine the association between participation in the NRCMS/NRSPI and six types of health outcomes.

**Results:**

Participation in the NRSPI was positively associated with some health outcomes, but the associations were relatively modest and were observed only for some specific age and household income groups. Participation in NRCMS was not associated with any health outcomes.

**Conclusions:**

The results provide limited evidence of the positive impact of social insurance on health among middle-aged and older adults in rural China. Thus, social insurance programs should be reformed to enhance their positive impact on health.

**Supplementary Information:**

The online version contains supplementary material available at 10.1186/s12889-020-09945-2.

## Background

To maintain the living standard of the elderly and reduce their risk of poverty and poor health in later life, establishing a universal social security system has become an important policy challenge in both developing and developed countries. This is particularly true of China, which is facing the serious problem of an aging population; the proportion of people aged 65 and older is expected to rise rapidly from 10.9% in 2017 to 26.3% in 2050 [1].

Correspondingly, an increasing number of studies have examined the impact of social insurance on health in many countries, including China. However, the results have generally been mixed. In terms of the impact of medical insurance on health, studies have obtained inconsistent findings for both developed countries [2–4] and developing countries [5–7]. For example, in the case of China, some studies found that medical insurance has a positive impact on health [8–13], while others failed to find any significant impact [14–16]; one study even found a negative impact [17]. Meanwhile, many studies have found public pensions to have a positive impact on health [7, 18, 19], with one study reporting the positive impact of pensions on health among rural residents in China [18]. However, one study found no significant impact in Korea [20].

In general, social insurance, which includes public pensions and medical insurance, may affect an individual’s health in several ways. First, both public pensions and medical insurance may increase the utilization of healthcare services and medical examinations, thus improving people’s health [21–23]. Medical insurance can reduce the out-of-pocket (OOP) costs of healthcare and the benefits of public pensions may directly increase household income, which can finance health expenditures. Second, both public pensions and medical insurance can have a positive impact on mental health by reducing uncertainty about future disposable income and health expenditures. Third, medical insurance may lead to problems of moral hazard, which may worsen an individual’s health [24]; insured individuals may be more inclined to engage in unhealthy behaviors such as smoking and excessive drinking. Fourth, exposure to the bureaucratic processes used to administer social insurance schemes can also impact health, especially in pensions, disability insurance, and worker compensation schemes [25–28]. Thus, the overall effect of social insurance on health should be evaluated using a large-scale dataset.

In the current study, we attempted to examine the impact of social insurance programs in China—the New Rural Social Pension Insurance (NRSPI) and the New Rural Cooperative Medical Scheme (NRCMS)—on the health of middle-aged and older adults in rural China, using three-wave longitudinal data from the China Health and Retirement Longitudinal Study (CHARLS).

In contrast with other countries, China’s social insurance schemes have been fragmented by the population registration system known as *Hukou* (e.g., rural *Hukou* and urban *Hukou*) since the planned economy period [13, 29, 30]. Although a public pension program for urban workers was inaugurated in the 1950s, the public pension scheme targeting rural *Hukou* residents was not established until the early 2000s. The NRSPI was formally launched in 2009 as the first pension insurance program to cover rural *Hukou* residents. Rural residents who are not covered by any other public pension insurance—such as urban employee basic pension insurance (UEBPI) or urban residents’ social pension insurance (URSPI)—can join the NRSPI.

Regarding medical insurance, its public scheme for urban workers was established in the 1950s, whereas in rural areas the Cooperative Medical Scheme (CMS), which was managed by the country’s communities and unsupported by the central government, was introduced in the 1950s [16]. The proportion of the population enrolled in CMS was as high as 90% in the late 1970s. However, with the collapse of the collective economy in the early 1980s, the coverage rate fell sharply to 5% in 1985. The NRCMS was introduced in 2003 to replace the CMS and now covers all rural *Hukou* individuals. The NRCMS is financed by individuals, rural communities, and local and central governments.

This study is expected to make three contributions to the literature on the impact of social insurance on health. First, based on three-wave longitudinal survey data, it addressed statistical issues such as the initial value effect (i.e., the effect of a variable’s initial value on its current value) and individual heterogeneity (i.e., an individual’s observed and unobserved attributes), and reverse causality. These problems have remained largely unsolved in previous studies, most of which have been cross-sectional. Second, unlike previous studies that concentrated on a specific type of scheme, this study compared both schemes’ impacts on health. Third, this study compared the results between different age and household income groups, a feature that previous studies have largely ignored. Notably, we compared the impact of the NRSPI on the health of middle-aged adults who had not started to receive pension benefits, with its impact on those aged 60 or above, unlike previous studies that only considered the latter [18].

## Methods

### Study sample

We used data obtained from the CHARLS, which is a nationwide longitudinal survey conducted by Peking University in representative regions of China in 2011, 2013, and 2015. The survey respondents comprised of individuals aged 45 years or older in the baseline survey. The CHARLS contains rich individual- and household-level information, such as a set of indices on health, demographic characteristics, family structure, house ownership, health behavior, and social participation, which were used in this study. The baseline wave included 17,708 individuals in 150 counties/districts and 450 villages/resident communities. The CHARLS is widely used in empirical studies on social insurance or health issues in China [31–34].

This study focused on rural *Hukou* residents aged 45 years or older in the baseline survey, who committed to at least one of two follow-up surveys. After excluding respondents who were missing key variables used in the statistical analysis, the total number of individuals whose data were used in this study was 32,808 (10,245 from 2011, 10,630 from 2013, and 11,933 from 2015). The sample used in the regression differed slightly depending on the model.

### Variables

Key independent variables were binary for participation in the NRSPI and NRCMS. As for health variables, we used six indices of health: (i) self-rated health (SRH), (ii) cognitive function (CF), (iii) mental health prospects (MH1), (iv) mental health at present (MH2), (v) no health issues affecting working capacity (NHP), and (vi) no disease. (i)–(iii) are continuous variables, while (v) and (vi) are binary variables. Regarding SRH and CF, we categorized them as *excellent* = 5, *very good* = 4, *good* = 3, *fair* = 2, and *poor* = 1, respectively. We constructed the binary variables of SRH and CF as 1 = *excellent*, 0 = *otherwise.* As for MH1, we categorized the answers to the question “How often do you feel hopeful about the future?” into *most or all of the time (5–7 days)* = 4, *occasionally or a moderate amount of the time (3–4 days)* = 3, *some or a little of the time (1–2 days)* = 2, and *rarely or none of the time (< one day)* = 1. As for MH2, we categorized the answers to the question “How often have you felt depressed recently?” in a revised order. We constructed a binary variable of MH1 or MH2 taking the value 1 when the value of MH1 or MH2 is equal to 4, and 0 otherwise. MH1 was part of the original questionnaire in the CHARLS, and was used for the first time in this study.

Regarding NHP, we constructed a binary variable for it by allocating 1 to the answer “no health problems at work” and 0 otherwise. We also constructed a binary variable of no disease by allocating 1 to those who answered that they had no disease diagnosed by doctors and 0 otherwise.

We considered the following as covariates, all of which are likely to have affected the association between social insurance and health and available from the CHARLS: (1) demographic factors including age, gender, and educational attainment (junior high school and lower, senior high school, and college or higher); (2) family factors including having a spouse or not, having living parents or not, number of family members, and living arrangement with children (residing together or apart); (3) house ownership; (4) health behavior (smoking and drinking); (5) social participation (participating in at least one of its seven types); (6) regions (east, central, west, and northeast), and (7) survey years (2011, 2013, and 2015). We used house ownership as a proxy for household income because using household income would substantially reduce the sample size due to missing variables. Housing assets share the largest portion (59.1%) of total household assets according to the 2013 China Household Income Project Survey, and it is positively correlated with household income [35].

The NRSPI benefit consists of two components: (i) the basic pension benefit fully funded by government subsidies, and (ii) the benefit corresponding to an individual’s contributions accumulated in a personal account. According to the CHARLS, the average NRSPI benefit in 2011 was 965 CNY per year, which was equivalent to 10.5% of per capita household income. Meanwhile, the NRCMS is a reimbursement system; its participants must first pay the total healthcare fee by themselves, and then seek reimbursement after submitting claims to their local governments. The NRCMS mostly covers inpatient and serious diseases, while most outpatients are excluded. The OOP rate is around 30%, which varies depending on the type of disease and region.

### Analytic strategy

As the benchmark, we considered the regression model to explain the health variable by participation in the NRSPI and NRCMS along with a set of covariates, X:

1 $$ {H}_i=a+\beta {NRSPI}_i+\gamma {NRCMS}_i+{\sum}_n{\delta}_n{X}_{ni}+{\varepsilon}_i, $$

where *i* denotes the individual and *ε* is an error term. If *ε* includes an individual-specific, time-invariant factor, heterogeneity problems may occur when these factors are not controlled for. To address this problem, we used the fixed-effects (FE) or random-effects (RE) model. We employed the *F* test, the Breusch and Pagan Lagrange multiplier test, and the Hausman specification test to compare the appropriateness of pooled ordinary least squares, FE, and RE models. As the results of these tests indicated that the FE model was the most appropriate, we employed it in this study.

We further considered an initial value problem [36–38]: health at time *t* might be affected by health at time *t* − 1. To address this problem, we considered a dynamic FE model, which includes health at time *t* − 1 as an explanatory variable. We further replaced all other explanatory variables at time *t* with those at time *t* − 1 to mitigate the reverse causality problem by allowing a one-wave (that is, two-year) lag from the explanatory variables to health [39, 40]. Overall, we estimated dynamic FE models with lagged explanatory variables using balanced panel data:

2 $$ {H}_{it}=a+\rho {H}_{it-1}+\beta {NRSPI}_{it-1}+\gamma {NRCMS}_{it-1}+{\sum}_n{\delta}_n{X}_{nit-1}+{u}_{it}, $$

where *t* and *t* − 1 denote a combination of survey years (2013 and 2011) or (2015 and 2013), and *u* is an error term. In the actual regression analysis, we estimated logistic regression models using a set of binary variables of health indices, rather than linear models. We estimated the regression models not only for the entire sample, but also for each age group (aged 45–59, 60–64, and 65 or above) and household income group (low, middle, and high) to examine heterogeneity.

## Results

### Descriptive analysis

**Fig. 1:**
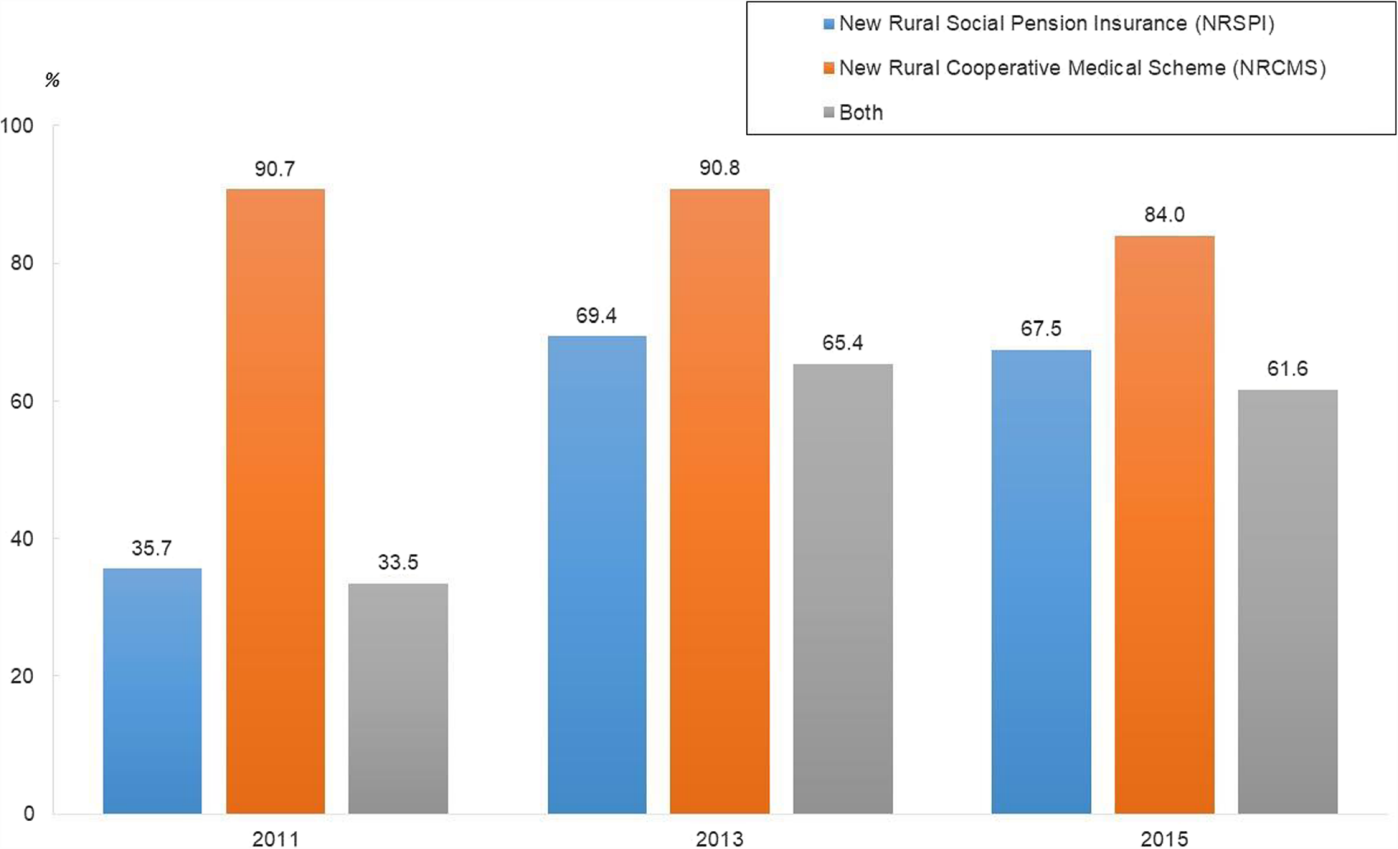
Participation rates in social insurance programs

Table 1 summarizes the key features of the study sample used in the statistical analysis. Figure 1 summarizes the participation rates of the NRSPI and NRCMS from 2011 to 2015. The participation rates for the NRSPI rose from 35.7% in 2011 to 69.4% in 2013 and 67.5% in 2015, indicating that the Chinese government succeeded in raising participation in public pensions for rural areas after introducing them in 2009. By comparison, the enrollment percentage in the NRCMS, which was introduced in 2003, remained high during the study period at approximately 90%. The proportion of participants in both the NRSPI and NRCMS rose from 33.5% in 2011 to 65.4% in 2013 and 61.6% in 2015, largely reflecting the increase in participation rates for the NRSPI.

**Table 1:** Key features of the study sample

	*N*	%		*N*	%
Demographic factors			Health behavior		
Gender			Smoking		
Men	15,678	(46.9)	No smoking	10,564	(31.6)
Women	17,751	(53.1)	Smoking in the past	14,207	(42.5)
Age (years)	*M*	59.3	Smoking currently	8658	(25.9)
	*SD*	(9.6)	Drinking		
Educational attainment			No drinking	25,139	(75.2)
Junior high school or lower	31,423	(94.0)	Drinking per week	6686	(20.0)
Senior high school	1791	(5.7)	Drinking per month	1604	(4.8)
College or higher	215	(0.3)	Social participation	15,277	(45.7)
Family factors			Regions		
Having a spouse	29,016	(86.8)	East	6285	(18.8)
Having living parents	4413	(16.9)	Central	6920	(20.7)
Living arrangement with children			West	19,355	(57.9)
Living together	18,597	(57.0)	Northeast	869	(2.6)
Living apart	14,211	(43.0)	Survey years		
Number of family members	*M*	3.47	2011	10,530	(31.5)
	*SD*	(1.7)	2013	10,864	(32.5)
House ownership	20,325	(60.8)	2015	12,035	(36.0)
*N*				33,429	

The age and number of family members are shown by mean values

Table 2 presents the unadjusted association between participation in social insurance programs and health outcomes, comparing health outcomes between participants and non-participants in the NRSPI and NRCMS using the entire sample. NRSPI participation was negatively associated with CF, MH2, and no disease, and positively associated with MH1. Meanwhile, participation in the NRCMS was negatively associated with all health outcomes except for MH2. It should be noted, however, that the comparisons in this table did not control for covariates and were not adjusted for potential biases related to cross-sectional comparisons. The results for each survey year (see Additional file 1) showed almost the same pattern as seen in the entire sample reported in Table 2.

**Table 2:** Unadjusted association between participation in social insurance programs and health outcomes^a^: total sample in 2011–2015

Proportion (%)	Participants	Non-participants	Difference		Participants		Non-participants		Total
	(a)	(b)	(a) – (b)	*t-*test	*N*	%	*N*	%	*N*
New Rural Social Pension Insurance (NRSPI)									
Self-rated health (SRH: 1–5)	2.13	2.12	0.01	0.440	22,124	(60.1)	14,669	(39.9)	36,793
Cognitive function (CF: 1–5)	1.83	1.86	−0.03	*p* < 0.001	22,601	(58.4)	16,118	(41.6)	38,719
Mental health in prospect (MH1: 1–4)	3.12	3.07	0.05	*p* < 0.001	22,274	(58.4)	15,869	(41.6)	38,143
Mental health at present (MH2: 1–4)	2.52	2.61	−0.09	*p* < 0.001	21,794	(58.4)	15,493	(41.6)	37,287
No health problem for working (NHP: 0–1)	0.66	0.67	−0.01	0.142	15,480	(58.9)	10,787	(41.1)	26,267
No disease (0–1)	0.27	0.30	−0.03	*p* < 0.001	24,434	(58.0)	17,679	(42.0)	42,113
New Rural Cooperative Medical Scheme (NRCMS)									
Self-rated health (SRH: 1–5)	2.12	2.22	−0.11	*p* < 0.001	32,516	(88.3)	4304	(11.7)	36,820
Cognitive function (CF: 1–5)	1.84	1.89	−0.06	*p* < 0.001	34,334	(88.6)	4405	(11.4)	38,739
Mental health in prospect (MH1: 1–4)	3.09	3.15	−0.06	*p* < 0.001	33,843	(88.7)	4317	(11.3)	38,160
Mental health at present (MH2: 1–4)	2.56	2.54	0.02	0.432	33,114	(88.8)	4190	(11.2)	37,304
No health problem for working (NHP: 0–1)	0.66	0.70	−0.04	*p* < 0.001	23,736	(90.3)	2542	(9.7)	26,278
No disease (0–1)	0.28	0.32	−0.04	*p* < 0.001	37,263	(88.4)	4912	(11.6)	42,175

^a^The higher the score, the better the health outcomes

### Regression analysis

The results of the regression models are summarized in Table 3, which reports the odds ratios (ORs) of reporting better health status, along with 95% confidence intervals (CIs), in response to participation in the NRSPI and NRCMS. Unlike the results based on the cross-sectional data reported in Table 2, there was no negative association with any health outcome.

**Table 3:** Estimated associations between participation in social insurance and health outcomes^a^

	OR	95% CI	*N*
New Rural Social Pension Insurance (NRSPI)			
Self-rated health (SRH)	1.06✝	(0.97, 1.18)	18,358
Cognitive function (CF)	1.30**	(1.10, 1.57)	21,714
Mental health prospects (MH1)	0.94	(0.92, 1.12)	20,002
Mental health at present (MH2)	1.07✝	(0.99, 1.17)	10,190
No health problem for working (NHP)	0.95	(0.85, 1.06)	11,878
No disease	1.01*	(1.00, 1.03)	21,776
New Rural Cooperative Medical Scheme (NRCMS)			
Self-rated health (SRH)	1.01	(0.98, 1.00)	18,358
Cognitive function (CF)	0.96	(0.77, 1.20)	21,714
Mental health prospects (MH1)	1.02	(0.91, 1.12)	20,002
Mental health at present (MH2)	1.07✝	(0.97, 1.08)	10,190
No health problem for working (NHP)	0.79	(0.65, 0.93)	11,878
No disease	0.95	(0.78, 1.16)	21,776

^a^Obtained from the dynamic fixed-effects ordered logistic or logistic models with lagged explanatory variables (controlled for covariates)

^*^ *p* < 0.05, ^**^ *p* < 0.05, ^✝^*p* < 0.1

Specifically, the table shows that participation in the NRSPI had positive and significant (*p* < 0.05) associations with CF (OR: 1.30, 95% CI: 1.10,1.57) and no disease (OR: 1.01, 95% CI: 1.00,1.03), while it had less significant but positive associations (*p* < 0.1) with SRH (OR: 1.06, 95% CI: 0.97,1.18) and MH2 (OR: 1.07, 95% CI: 0.99,1.17). The positive association between participation in the NRCMS and health was more limited, as it only had a modest association with MH2 (OR: 1.07, 95% CI: 0.97,1.08).

Tables 4 and 5 summarize the results obtained from separate estimations by age and household income, respectively. Table 4 indicates that participation in the NRSPI had modestly positive associations with SRH, CF, and MH2 (*p* < 0.1) among those aged 45–59, while the results were mixed among older age groups; participation in the NRSPI was negatively associated with NH1 and NHP (*p* < 0.05) among those aged 65 or above. In general, the results for the NRCMS were more mixed, in such a way that no clear pattern emerged.

**Table 4:** Estimated associations between participation in social insurance and health outcomes by age group^a^

	Aged 45–59		Aged 60–64		Aged 65 or above	
	OR	95% CI	OR	95% CI	OR	95% CI
New Rural Social Pension Insurance (NRSPI)						
Self-rated health (SRH)	1.12^✝^	(0.98, 1.28)	1.19	(0.95, 1.49)	0.92	(0.77, 1.10)
Cognitive function (CF)	1.24^✝^	(0.99, 1.54)	1.98^**^	(1.33, 2.94)	1.12	(0.79, 1.60)
Mental health prospects (MH1)	1.00	(0.89, 1.11)	0.95	(0.79, 1.13)	0.85 ^*^	(0.75, 0.98)
Mental health at present (MH2)	1.11^✝^	(0.98, 1.24)	1.07	(0.88, 1.29)	1.05	(0.88, 1.26)
No health problem for working (NHP)	0.99	(0.86, 1.14)	1.08	(0.84, 1.38)	0.77 ^*^	(0.59, 1.00)
No disease	1.12	(0.92, 1.36)	0.75^✝^	(0.54, 1.05)	1.09	(0.81, 1.48)
New Rural Cooperative Medical Scheme (NRCMS)						
Self-rated health (SRH)	0.88	(0.73, 1.28)	0.90	(0.66, 1.22)	0.89	(0.71, 1.10)
Cognitive function (CF)	0.90	(0.66, 1.21)	0.66^*^	(0.41, 1.08)	1.37	(0.87, 2.17)
Mental health in prospect (MH1)	1.00	(0.86, 1.17)	1.15	(0.90, 1.46)	0.99	(0.82, 1.18)
Mental health at present (MH2)	0.97	(0.82, 1.21)	0.88	(0.68, 1.13)	1.07	(0.86, 1.33)
No health problem for working (NHP)	0.66^***^	(0.54, 0.82)	1.09	(0.76, 1.56)	1.00	(0.70, 1.43)
No disease	0.85	(0.65, 1.12)	1.58^*^	(1.00, 2.49)	0.83	(0.56, 1.21)

^a^Obtained from the dynamic fixed-effects ordered logistic or logistic models with lagged explanatory variables (controlled for covariates)

^***^ *p* < 0.001, ^**^ *p* < 0.01, ^*^ *p* < 0.05, ^✝^*p* < 0.1

**Table 5:** Estimated associations between participation in social insurance and health outcomes by household income group^a^

	Low		Middle		High	
	OR	95% CI	OR	95% CI	OR	95% CI
New Rural Social Pension Insurance (NRSPI)						
Self-rated health (SRH)	1.28^*^	(1.02, 1.59)	0.99	(0.79, 1.25)	1.16	(0.94, 1.45)
Cognitive function (CF)	2.04^***^	(1.29, 3.00)	0.98	(0.64, 1.51	1.30	(0.91, 1.88)
Mental health prospects (MH1)	1.00	(0.84, 1.20)	0.87	(0.72, 1.04)	0.86	(0.72, 1.04)
Mental health at present (MH2)	1.18^✝^	(0.97, 1.43)	0.90	(0.75, 1.10)	1.06	(0.88, 1.28)
No health problem for working (NHP)	1.07	(0.76, 1.52)	0.98	(0.77, 1.24)	0.94	(0.74, 1.20)
Non-disease	1.28^*^	(1.02, 1.59)	0.96	(0.67, 1.39)	1.36^✝^	(0.96, 1.92)
New Rural Cooperative Medical Scheme (NRCMS)						
Self-rated health (SRH)	0.88	(0.64, 1.20)	0.86	(0.63, 1.17)	0.90	(0.70, 1.17)
Cognitive function (CF)	1.05	(0.60, 1.83)	0.87	(0.50, 1.50)	0.82	(0.54, 1.24)
Mental health in prospect (MH1)	1.27^✝^	(0.96, 1.65)	0.96	(0.75, 1.21)	0.87	(0.70, 1.09)
Mental health at present (MH2)	1.03	(0.77, 1.36)	1.01	(0.78, 1.32)	0.80	(0.64, 1.00)
No health problem for working (NHP)	1.29	(0.80, 2.08)	0.91	(0.66, 1.27)	0.83	(0.60, 1.13)
Non-disease	0.88	(0.64, 1.20)	0.80	(0.46, 1.38)	1.24	(0.83, 1.86)

^a^Obtained from the dynamic fixed-effects ordered logistic or logistic models with lagged explanatory variables (controlled for covariates)

^***^ *p* < 0.001, ^*^ *p* < 0.05, ^✝^*p* < 0.1

Table 5 compares the association between participation in the NRSPI/NRCMS and health by household income group. The most noticeable finding was that participation in the NRSPI was positively associated with SRH, CF, and no disease (*p* < 0.05), and to a lesser extent MH2 (*p* < 0.1) among the low-income group. Excluding these combinations, neither the NRSPI nor NRCMS were associated with any health outcome.

## Discussion

We examined how two social insurance programs, the NRSPI and NRCMS, were associated with health among middle-aged and older adults in rural China. Our longitudinal regression analysis, based on dynamic FE models with lagged explanatory variables, indicated that participation in the NRSPI had positive associations with some health outcomes, but that the associations were relatively modest and were observed only for some specific age and household income groups. Meanwhile, participation in the NRCMS was not associated with any health outcome. These results are generally in line with the mixed results from previous studies in China [8–17], which did not fully control for statistical biases. Regarding the association between the NRSPI and health, our results contrasted with those of Cheng et al. [18], who reported more positive results using the CHARLS data. However, there are three differences between their study and ours. First, we used three-wave longitudinal survey data from 2011 to 2015, whereas they only used two-wave (2008/2009 vs. 2010/2011) data. Second, we employed dynamic FE models with lagged explanatory variables, whereas they employed the usual FE and FE models with instrumental variables. Third, we included individuals aged 45–59 in the sample, whereas they focused solely on those aged 60 and above. As for the association between medical insurance and health in China, most previous studies reported that the NRCMS increased the probability of healthcare utilization and improved health [12, 21, 30, 41]. These previous studies used data from earlier periods (such as 2000–2008) than ours (2011–2013), suggesting that the impact of the NRCMS on health should be analyzed from both short-term and long-term perspectives.

Our estimation results indicated a positive, albeit modest, impact of the NRSPI on health among rural *Hukou* residents, particularly among those aged 45–60 and from low-income groups. These results suggest that the NRSPI may help have a positive, albeit modest, and indirect effect on people’s health in rural areas by reducing uncertainty about income after retirement, especially among low-income groups.

Meanwhile, no positive impact of the NRSPI on health was consistently observed among those aged 60 and older. The adaptation hypothesis can explain why individuals are more likely to take pension benefits for granted when they continue to receive them over a period of time. The results were also likely attributable to low levels of NRSPI benefits, which were below the poverty line in rural areas. According to the CHARLS, the individual average NRSPI pension benefit in 2011 and 2013 was 965 CNY per year, compared to the 2300 CNY per year standard poverty line in rural areas since 2011 [42].

We also found that the NRCMS had no impact on most health outcomes, pointing to limitations in this program compared to the public medical insurance covering urban workers, the Urban Employee Basic Medical Insurance (UEBMI). Although the percentage of OOP for healthcare expenditure is regulated to be 30% in both the NRCMS and UEBMI, most outpatients are excluded from the NRCMS, and fewer types of fatal diseases are covered by it. Moreover, the reinvestment system in the NRCMS may make low-income individuals more inclined to avoid using healthcare services. These factors seem to explain the limited impact of the NRCMS, which was expected to improve the health outcomes of rural *Hukou* residents.

Based on the results of this study, the Chinese government should increase NRSPI benefits to support income conditions for rural *Hukou* residents, particularly those with low incomes. The government should also reform the NRCMS to reduce the payment of healthcare fees by rural *Hukou* residents and cover outpatients, as well as more types of diseases.

## Conclusions

We conclude that the NRSPI is positively associated with some health outcomes, but that the associations are relatively modest and observed only in some specific age and household income groups. Participation in the NRCMS has no association with any health outcomes. These results provide limited evidence of the positive impact of social insurance on health among middle-aged and older adults in rural China.

This study has several limitations. First, although we used dynamic FE models with lagged explanatory variables, we could not identify the causation from social insurance to health, which should be investigated in a more in-depth analysis. Second, because we focused on individuals aged 45 or older due to data limitations, future studies should expand the analysis to include younger individuals. Third, the question of why the association with social insurance was observed for selected types of health outcomes remains unanswered.

Despite these limitations, we believe that the current study, which took full advantage of longitudinal data, provides new insights for understanding the association between social insurance and health. We also expect the Chinese experience of reforming social insurance policy for rural *Hukou* residents to provide valuable lessons for developing countries that are also looking to establish or reform their own social insurance schemes.

## Supplementary Information

**Additional file 1.**

## Data Availability

The dataset that was used in this study, the China Health and Retirement Longitudinal Study (CHARLS), is publicly available (http://charls.pku.edu.cn/en).

## Abbreviations

CF: Cognitive function
CHARLS: China Health and Retirement Longitudinal Study
CI: Confidence interval
CMS: Cooperative Medical Scheme
FE: Fixed-effects
MH1: Mental health prospects
MH2: Mental health at present
NHP: No health problem for working
NRCMS: New Rural Cooperative Medical Scheme
NRSPI: New Rural Social Pension Insurance
OOP: Out-of-pocket
OR: Odds ratio
RE: Random-effects
SRH: Self-rated health
UEBMI: Urban Employee Basic Medical Insurance

## References

[1] OECD (2019). Pensions at a glance 2019.

[2] Courtin E, Kim S, Song S, Yu W, Muennig P (2020). Can social policies improve health? A systematic review and meta-analysis of 38 randomized trials. Milbank Q.

[3] Finkelstein A, McKnight R (2008). What did Medicare do? The initial impact of Medicare on mortality and out of pocket medical spending. J Public Econ.

[4] Jensen R, Richer K (2004). The health implications of social security failure: evidence from the Russian pension crisis. J Public Econ.

[5] Case A, Wise D (2004). Does money protect health? Evidence from South Africa pensions. Perspectives on the economics of ageing.

[6] Card D, Dobkin C, Maestas N (2009). Does Medicare save lives?. Q J Econ.

[7] Schatz E, Gómez-Olivé X, Ralston M, Menken J, Tollman S (2012). The impact of pensions on health and wellbeing in rural South Africa: does gender matter?. Soc Sci Med.

[8] Cheng L, Liu H, Zhang Y, Shen K, Zeng Y (2015). The impact of health insurance on health outcomes and spending of the elderly: evidence from China’s new cooperative medical scheme. Health Econ.

[9] Gu L, Feng H, Jin J (2017). Effects of medical insurance on the health status and life satisfaction of the elderly. Iran J Public Health.

[10] Huang F, Gan L (2010). Excessive demand or effective demand? Empirical analysis of urban elderly health and medical insurance. J Econ Res.

[11] Huang F, Wu C (2009). Impact of China’s medical insurance on urban elderly mortality rate. Nankai Econ Res.

[12] Jiang K, You D, Li Z, Wei W, Mainstone M (2018). Effects of rural medical insurance on chronically ill patients’ choice of the same hospital again in rural northern China. Int J Environ Res Public Health.

[13] Qin X, Pan J, Liu GG (2014). Does participating in health insurance benefit the migrant workers in China? An empirical investigation. China Econ Rev.

[14] Bai CE, Wu B (2014). Health insurance and consumption: evidence from China’s new cooperative medical scheme. J Comp Econ.

[15] Chen Y, Jin GZ (2012). Does health insurance coverage lead to better health and educational outcomes? Evidence from rural China. J Health Econ.

[16] Lei X, Lin W (2009). The new cooperative medical scheme in rural China: does more coverage mean more service and better health?. Health Econ.

[17] Luo C (2008). Study on the health differences of urban residents and medical expenditure behavior. Stud Finance Econ.

[18] Cheng LG, Liu H, Zhang Y, Zhao Z (2018). The health implications of social pensions: evidence from China’s new rural pension scheme. J Comp Econ.

[19] Galiani S, Gertler P, Bando R (2016). Non-contributory pensions. Labour Econ.

[20] Pak TY (2019). Social protection for happiness? The impact of social pension reform on subjective well-being of the Korean elderly. J Policy Model.

[21] Pan J, Lei X, Liu GG (2016). Health insurance and health status: exploring the causal effect from a policy intervention. Health Econ.

[22] Cuong VN (2012). The impact of voluntary health insurance on health care utilization and out of pocket payment: new evidence for Vietnam. Health Econ.

[23] Wagstaff A, Lindelow M, Gao J, Xu L, Qian JC (2009). Extending health insurance to the rural population: an evaluation of China’ cooperative medical scheme. J Health Econ.

[24] Arrow KJ, John WP, Richard JZ (1984). The economics of agency. Principals & agents: the structure of business.

[25] Bartys S, Frederiksen P, Bendix T, Burton K (2017). System influences on work disability due to low back pain: an international evidence synthesis. Health Policy.

[26] Esser I, Palme J (2010). Do public pensions matter for health and wellbeing among retired persons? Basic and income security pensions across 13 Western European countries. Int J Soc Welf.

[27] Grant G, Studdert DM (2009). Poisoned chalice? A critical analysis of the evidence linking personal injury compensation processes with adverse health outcomes. Melb U L Rev.

[28] Weathers RR, Stegman M (2012). The effect of expanding access to health insurance on the health and mortality of social security disability insurance beneficiaries. J Health Econ.

[29] Ma X (2016). Public medical insurance system reform and the determinants of participation to the medical insurance systems in the ageing China. J Popul Probl.

[30] Meng Q, Fang H, Liu X, Yuan B, Xu J (2015). Consolidating the social health insurance schemes in China: towards an equitable and efficient health system. Lancet..

[31] Ning M, Gong J, Zheng XH, Zhuang J (2016). Does new rural pension scheme decrease elderly labor supply? Evidence from CHARLS. China Econ Rev.

[32] Luo HQ, Ren XH, Li JJ, Wu K, Wang YX, Chen Q, Li NX (2020). Association between obesity status and successful aging among older people in China: evidence from CHARLS. BMC Public Health.

[33] Ma X, Piao X, Oshio T (2020). Impact of social participation on health among middle-aged and elderly adults: evidence from longitudinal survey data in China. BMC Public Health.

[34] Lu B, Yang MX, Cumming RG, Stanaway FF. Falls and impact on disability and healthy life expectancy in China: evidence from the China health and retirement longitudinal survey (CHARLS). China Econ Rev. 2020.

[35] Knight J, Li S, Wan H, Sicular T, Sato H, Li S, Yue X (2017). The increased trend of wealth inequality in China. The latest change of income distribution in China.

[36] Contoyannis P, Jones A, Rice N (2004). The dynamics of health in the British household panel survey. J Appl Econ.

[37] Wooldridge J (2005). Simple solutions to the initial conditions problem in dynamic, nonlinear panel data models with unobserved heterogeneity. J Appl Econ.

[38] Wooldridge J (2020). Econometric analysis of cross section and panel data.

[39] Rothman KJ, Poole C (1985). Science and policy making. Am J Public Health.

[40] Green WH (2012). Econometric analysis.

[41] Wang H, Yip W, Zhang L, Hsiao WC (2009). The impact of rural mutual health care on health status: evaluation of a social experiment in rural China. Health Econ.

[42] Shu L (2018). The effects of the new rural social pension insurance program on the retirement and labor supply decision in China. J Econ Ageing.

