# Supplementary material for: The impact of social insurance on health among middle-aged and older adults in rural China: a longitudinal study using a three-wave nationwide survey
Source: BMC Public Health. 2020 Dec 1;20:1842. doi: 10.1186/s12889-020-09945-2 (PMC7709307; doi:10.1186/s12889-020-09945-2)
Supplement: Supplementary file 1 — Additional file 1. [file 12889_2020_9945_MOESM1_ESM.docx]

**Unadjusted association between participation in social insurance programs and health outcomes in each survey year** (see Table 2 in main text)

**(1) 2011**

| Proportion (%) | Participants | Non-participants | Difference | | Participants | | Non-participants | | Total |
| --- | --- | --- | --- | --- | --- | --- | --- | --- | --- |
|  | (a) | (b) | (a) – (b) | *t-*test | *N* | % | *N* | % | *N* |
| New Rural Social Pension Insurance (NRSPI) | |  |  |  |  |  |  |  |  |
| Self-rated health (SRH: 1–5) | 2.14 | 2.06 | 0.08 | *p* < 0.001 | 3393 | (35.8) | 6094 | (64.2) | 9487 |
| Cognitive function (CF: 1–5) | 1.82 | 1.84 | -0.02 | 0.236 | 4375 | (35.9) | 7811 | (64.1) | 12186 |
| Mental health prospects (MH1: 1–4) | 3.02 | 2.93 | 0.09 | *p* < 0.001 | 4315 | (35.9) | 7707 | (64.1) | 12022 |
| Mental health at present (MH2: 1–4) | 2.79 | 2.73 | 0.06 | *p* < 0.006 | 4248 | (35.9) | 7586 | (64.1) | 11834 |
| No health problem for working (NHP: 0–1) | 0.72 | 0.65 | 0.07 | *p* < 0.001 | 3177 | (36.5) | 5519 | (63.5) | 8696 |
| No disease (0–1) | 0.31 | 0.31 | 0.00 | 0.897 | 4758 | (35.7) | 8588 | (64.3) | 13346 |
| New Rural Cooperative Medical Scheme (NRCMS) | |  |  |  |  |  |  |  |  |
| Self-rated health (SRH: 1–5) | 2.08 | 2.15 | -0.07 | 0.037 | 8600 | (90.6) | 893 | (9.4) | 9493 |
| Cognitive function (CF: 1–5) | 1.83 | 1.86 | -0.03 | 0.267 | 11092 | (91.0) | 1101 | (9.0) | 12193 |
| Mental health prospects (MH1: 1–4) | 2.96 | 2.96 | 0.00 | 0.992 | 10946 | (91.0) | 1083 | (9.0) | 12029 |
| Mental health at present (MH2: 1–4) | 2.76 | 2.69 | 0.07 | 0.103 | 10779 | (91.0) | 1062 | (9.0) | 11841 |
| No health problem for working (NHP: 0–1) | 0.68 | 0.70 | -0.02 | 0.201 | 8028 | (92.3) | 669 | (7.7) | 8697 |
| No disease (0–1) | 0.31 | 0.33 | -0.02 | 0.040 | 12114 | (90.7) | 1243 | (9.3) | 13357 |

**(2) 2013**

| Proportion (%) | Participants | Non-participants | Difference | | Participants | | Non-participants | | Total |
| --- | --- | --- | --- | --- | --- | --- | --- | --- | --- |
|  | (a) | (b) | (a) – (b) | *t-*test | *N* | % | *N* | % | *N* |
| New Rural Social Pension Insurance (NRSPI) | |  |  |  |  |  |  |  |  |
| Self-rated health (SRH: 1–5) | 2.11 | 2.10 | 0.01 | 0.010 | 9264 | (69.6) | 4053 | (30.4) | 13317 |
| Cognitive function (CF: 1–5) | 1.84 | 1.90 | -0.06 | *p* < 0.001 | 8791 | (69.8) | 3809 | (30.2) | 12600 |
| Mental health prospects (MH1: 1–4) | 3.18 | 3.15 | 0.03 | *p* < 0.001 | 8656 | (69.9) | 3736 | (30.1) | 12392 |
| Mental health at present (MH2: 1–4) | 2.38 | 2.43 | -0.05 | 0.034 | 8494 | (69.9) | 3662 | (30.1) | 12156 |
| No health problem for working (NHP: 0–1) | 0.63 | 0.69 | -0.06 | *p* < 0.001 | 6178 | (70.3) | 2611 | (29.7) | 8789 |
| No disease (0–1) | 0.28 | 0.31 | -0.03 | *p* < 0.001 | 9676 | (69.4) | 4267 | (30.6) | 13943 |
| New Rural Cooperative Medical Scheme (NRCMS) | |  |  |  |  |  |  |  |  |
| Self-rated health (SRH: 1–5) | 2.12 | 2.23 | -0.11 | *p* < 0.001 | 12118 | (90.9) | 1208 | (9.1) | 13326 |
| Cognitive function (CF: 1–5) | 1.86 | 1.88 | -0.02 | 0.281 | 11486 | (91.1) | 1120 | (8.9) | 12606 |
| Mental health prospects (MH1: 1–4) | 3.20 | 3.23 | -0.03 | 0.370 | 11301 | (91.2) | 1096 | (8.8) | 12397 |
| Mental health at present (MH2: 1–4) | 2.40 | 2.37 | 0.03 | 0.541 | 11088 | (91.2) | 1073 | (8.8) | 12161 |
| No health problem for working (NHP: 0–1) | 0.65 | 0.70 | -0.05 | *p* < 0.001 | 8108 | (92.2) | 685 | (7.8) | 8793 |
| No disease (0–1) | 0.28 | 0.34 | -0.06 | *p* < 0.001 | 12679 | (90.8) | 1292 | (9.2) | 13971 |

**(3) 2015**

| Proportion (%) | Participants | Non-participants | Difference | | Participants | | Non-participants | | Total |
| --- | --- | --- | --- | --- | --- | --- | --- | --- | --- |
|  | (a) | (b) | (a) – (b) | *t-*test | *N* | % | *N* | % | *N* |
| New Rural Social Pension Insurance (NRSPI) | |  |  |  |  |  |  |  |  |
| Self-rated health (SRH: 1–5) | 2.15 | 2.18 | -0.03 | 0.050 | 9467 | (67.7) | 4522 | (32.3) | 13989 |
| Cognitive function (CF: 1–5) | 1.82 | 1.87 | -0.05 | *p* < 0.001 | 9435 | (67.7) | 4498 | (32.3) | 13933 |
| Mental health prospects (MH1: 1–4) | 3.10 | 3.09 | 0.01 | 0.012 | 9303 | (67.8) | 4426 | (32.2) | 13729 |
| Mental health at present (MH2: 1–4) | 2.53 | 2.55 | -0.02 | 0.480 | 9052 | (68.1) | 4245 | (31.9) | 13297 |
| No health problem for working (NHP: 0–1) | 0.65 | 0.69 | -0.04 | 0.004 | 6125 | (69.7) | 2657 | (30.3) | 8782 |
| No disease (0–1) | 0.25 | 0.28 | -0.03 | *p* < 0.001 | 10000 | (67.5) | 4824 | (32.5) | 14824 |
| New Rural Cooperative Medical Scheme (NRCMS) | |  |  |  |  |  |  |  |  |
| Self-rated health (SRH: 1–5) | 2.14 | 2.25 | -0.11 | *p* < 0.001 | 11798 | (84.3) | 2203 | (15.7) | 14001 |
| Cognitive function (CF: 1–5) | 1.82 | 1.92 | -0.10 | *p* < 0.001 | 11756 | (84.3) | 2184 | (15.7) | 13940 |
| Mental health prospects (MH1: 1–4) | 3.10 | 3.21 | -0.11 | *p* < 0.001 | 11596 | (84.4) | 2138 | (15.6) | 13734 |
| Mental health at present (MH2: 1–4) | 2.54 | 2.53 | 0.02 | 0.473 | 11247 | (84.6) | 2055 | (15.4) | 13302 |
| No health problem for working (NHP: 0–1) | 0.66 | 0.71 | -0.05 | *p* < 0.001 | 7600 | (86.5) | 1188 | (13.5) | 8788 |
| No disease (0–1) | 0.25 | 0.30 | -0.05 | *p* < 0.001 | 12470 | (84.0) | 2377 | (16.0) | 14847 |
